# Hepatitis C virus infection in Brazilian long-distance truck drivers

**DOI:** 10.1186/1743-422X-7-205

**Published:** 2010-08-27

**Authors:** Nara R Freitas, Sheila A Teles, Marcos A Matos, Carmen LR Lopes, Nádia RS Reis, Márcia P Espírito-Santo, Elisabeth Lampe, Regina MB Martins

**Affiliations:** 1 Instituto de Patologia Tropical e Saúde Pública, Universidade Federal de Goiás (UFG), Goiás, Brazil; 2 Faculdade de Enfermagem, UFG, Goiás, Brazil; 3 Instituto Oswaldo Cruz, Fundação Oswaldo Cruz, Rio de Janeiro, Brazil

## Abstract

Hepatitis C virus (HCV) infection is a global public health problem. Long-distance truck drivers live apart from their family for long periods of time, a lifestyle that favors at-risk behaviors such as unprotected sex with multiple partners and illicit drug use. As data concerning HCV infection in this population are still rare, this paper aims to investigate the prevalence, genotypes/subtypes, and the factors associated with HCV infection in long-distance truck drivers in Brazil. A cross-sectional survey was carried out with 641 Brazilian long-truck drivers who were recruited at a major truck stop located at kilometer 1,296 of the BR-153 highway, which is considered to be one of the longest roads in Brazil. All individuals were interviewed, and their serum samples were tested for the presence of antibodies to HCV (anti-HCV) by ELISA and immunoblot. Anti-HCV positive samples were tested for HCV RNA by PCR amplification of the 5' NC and NS5B regions and were genotyped using the LiPA assay and nucleotide sequencing, respectively. Factors associated with HCV infection were identified with logistic regression. The prevalence of HCV infection was 1.4% (95% CI: 0.7-2.8). History of blood transfusion, sharing of personal hygiene tools, illicit drug use and HBV status were factors independently associated with HCV infection in the study population. HCV RNA was detected in 8/9 anti-HCV positive samples, in which genotypes 1 (n = 3), 2 (n = 2), and 3 (n = 3) were determined by LiPA. Using phylogenetic tree analysis of the NS5B region, subtypes 1a (n = 1), 1b (n = 2), 2b (n = 2) and 3a (n = 3) were identified. These data show that the prevalence of HCV infection among Brazilian truck drivers was similar to that observed for the general population. History of blood transfusion, sharing of personal hygiene tools, illicit drug use and HBV status were predictors of HCV infection. The HCV genotypes/subtypes identified in the study population are consistent with those circulating in Brazil.

## Findings

Hepatitis C virus (HCV) infection is a global public health problem. Approximately 130-170 million individuals are thought to be infected worldwide [1]. On average, 80% of acutely infected individuals develop a chronic infection. The principal long-term complications of chronic hepatitis C are cirrhosis and hepatocellular carcinoma [2]. HCV is characterized by a high degree of genetic heterogeneity. Phylogenetic analysis of full-length or partial sequences of HCV isolates has led to the identification of six genotypes (1 to 6), each comprising multiple subtypes (designated a, b, c, etc) [3]. These genotypes and subtypes have distinct geographical distributions, and information on their distribution is needed to perform effective molecular and epidemiological HCV surveillance [4,5].

Long-distance truck drivers live apart from their family for long periods of time, a lifestyle that favors at-risk behaviors such as unprotected sex with multiple partners, including commercial sex workers, and illicit drug use, which have been demonstrated to be predictors of HCV, hepatitis B virus (HBV), and human immunodeficiency virus (HIV) infections [6-9]. In spite of these risk factors, there have been few investigations on HCV infection in this population [9-12]. In a previous study, an overall HBV infection prevalence of 18.9% was found among long-distance truck drivers in Brazil. Length of profession longer than 20 years, time away from home lasting more than 15 days and a history of sexual transmitted infections (STIs) were associated with HBV infection [13]. However, no data are available on HCV infection in this highly mobile population in Brazil and more generally in Latin America. Thus, the aim of the present study was to investigate the prevalence, genotypes/subtypes, and the factors associated with HCV infection in long-distance truck drivers in Brazil.

**Figure 1:**
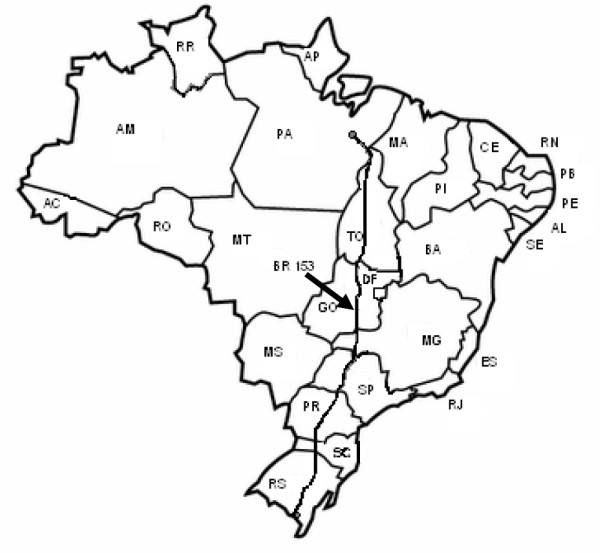
**Localization of the BR-153 highway in Brazil**.

A cross-sectional study was carried out in a population of long-distance truck drivers in Brazil that had been previously examined for HBV epidemiological status [13]. From October 2005 to October 2006, truck drivers were recruited once per month (on a Saturday or a Monday, during the morning or afternoon) at a major truck stop located at kilometer 1,296 of the BR-153 highway in Goiânia City, Central Brazil. This highway is considered to be one of the longest roads in Brazil (3,566.3 km), spanning the country from south to north (Figure 1). A total of 771 long-distance truck drivers from different regions of Brazil who stopped to refuel, eat and rest at this stop were invited to take part in the study. Of these, 641 agreed to participate, and informed consent was obtained from all. There was no statistical difference based on socio-demographic characteristics between the participants and those who were unwilling to participate (data not shown). The protocol used in the present study was approved by the Ethical Committee of the Materno Infantil Hospital in Goiânia city, Goiás state. The participants were interviewed to collect socio-demographic data and possible risk factors for HCV infection. Blood samples were collected from all participants, and sera were stored at -20°C.

All serum samples were tested using an enzyme-linked immunosorbent assay (ELISA) for the presence of antibodies to HCV (anti-HCV) (Hepanostika Ultra, Biomedical, Shanghai, China). Anti-HCV-positive samples were retested by an immunoblot (Bioblot HCV, Biokit, Barcelona, Spain) and submitted to RNA detection by a nested PCR with primers complementary to the conserved area of the 5' NC region of HCV [14]. A line probe assay (Inno-LiPA HCV II, Innogenetics, Ghent, Belgium) was used to determine the genotype of the isolates using amplicons of the 5' NC region. Nucleotide sequence analysis was performed after a semi-nested PCR amplification of the NS5B region [15].

Prevalence and 95% confidence intervals (95% CI) were calculated. Fisher's exact test was used to evaluate association between variables and HCV infection (defined as positive for anti-HCV). These, estimated by the odds ratio in univariate analysis, were further analyzed with a stepwise logistic regression model. Statistical significance was assessed at the 0.05 probability level in all analyses. Statistical evaluations were performed using SPSS, version 11.0 (SPSS Inc., Chicago, US, 1999).

The study group of 641 Brazilian long-distance truck drivers was composed mainly of males (99.2%). The mean age was 40.6 ± 10.1 years. The majority of the participants were white (69.9%), married (77%), had received eight years or less of formal education (fundamental education in Brazil) (68.8%) and were truck drivers for more than 10 years (62.6%). More than half of the group (51.9%) reported a monthly income of US$ 750 or less. Ten truck drivers were found to be anti-HCV positive by ELISA. Of these, nine were subsequently confirmed as positive by immunoblot, resulting in an anti-HCV prevalence of 1.4% (95% CI: 0.7-2.8).

Table 1 presents the univariate and multivariate analyses of the factors associated with HCV infection in long-distance truck drivers in Brazil. In the univariate analysis, sharing of personal hygiene tools, illicit drug use and HBV status were predictors of HCV infection and history of blood transfusion showed an association with a borderline *P *value. These factors were independent associated with HCV infection in a multivariate analysis.

**Table 1:** Factors associated with HCV infection in Brazilian long-distance truck drivers

	HCV		**OR**^**b**^		**OR**_**adjusted**_^**d **^	
Variables	**Pos/Total**^**a**^	(%)	**(95% CI)**^**c**^	*P*	(95% CI)	*P*
Age						
≤ 40 years	3/330	(0.9)	1.0			
> 40 years	6/306	(2.0)	2.2 (0.5-8.8)	0.32		
Duration of profession						
≤ 10 years	1/236	(0.4)	1.0			
11-20 years	5/200	(2.5)	6.0 (0.7-137.4)	0.10		
> 20 years	3/200	(1.5)	3.6 (0.3-90.0)	0.34		
Days away from home						
≤ 15	1/124	(0.8)	1.0			
> 15	8/512	(1.6)	1.9 (0.2-15.7)	1.00		
Blood transfusion						
No	6/560	(1.1)	1.0		1.0	
Yes	3/62	(4.8)	4.7 (1.1-19.2)	0.05	6.4 (1.3-32.3)	0.02
Sharing of personal hygiene tools						
No	5/543	(0.9)	1.0		1.0	
Yes	4/87	(4.6)	5.2 (1.4-19.7)	0.03	5.0 (1.1-22.3)	0.03
Illicit drug use						
No	5/527	(0.9)	1.0		1.0	
Yes	4/81	(4.9)	5.4 (1.4-20.6)	0.02	6.8 (1.4-32.0)	0.01
Tattoo/piercing						
No	8/582	(1.4)	1.0			
Yes	1/54	(1.9)	1.3 (0.2-11.0)	0.55		
Incarceration						
No	9/577	(1.6)				
Yes	0/57	(0.0)	-	1.00		
Sex with drug user						
No	6/410	(1.5)	1.0			
Yes	1/61	(1.6)	1.1 (0.1-9.5)	1.00		
Sex with a sex worker						
No	4/287	(1.4)	1.0			
Yes	5/349	(1.4)	1.0 (0.3-3.8)	1.00		
Sex with man						
No	6/616	(1.5)				
Yes	0/20	(0.0)	-	1.00		
Condon use during the last sexual intercourse						
Yes	4/307	(1.3)	1.0			
No	5/320	(1.6)	1.2 (0.3-4.5)	1.00		
Sexually transmitted infections						
No	5/396	(1.3)	1.0			
Yes	3/219	(1.4)	1.1 (0.3-4.6)	1.00		
Number of sexual partner (last 6 months)						
≤ 1	5/365	(1.4)	1.0			
2-5	2/191	(1.0)	0.8 (0.1-4.5)	1.00		
> 5	2/66	(3.0)	2.2 (0.3-13.4)	0.29		
HBV status						
Negative	4/519	(0.8)	1.0		1.0	
Positive	5/117	(4.3)	5.8 (1.5-22.0)	0.01	5.0 (1.2-20.1)	0.02

^a^The denominator represents the number of males who answered the question.

^b^OR, odds ratio.

^c^CI, confidence interval.

^d ^adjusted by age, blood transfusion, sharing of personal hygiene tools, illicit drug use and HBV status.

**Figure 2:**
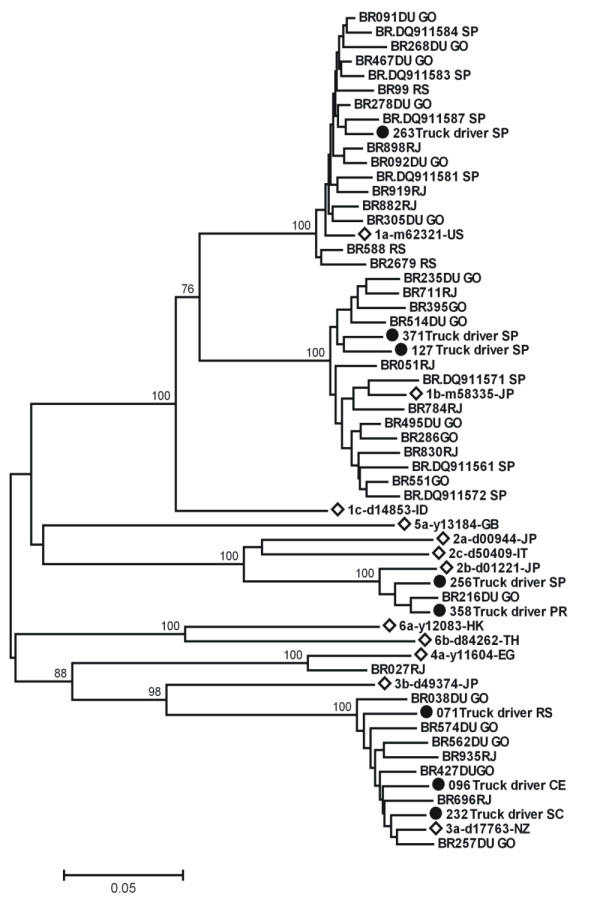
**Phylogenetic tree analysis of the NS5B region (340 nt) of HCV isolates**. The tree includes 8 sequences from truck drivers in Brazil, 12 reference sequences of genotypes 1 to 6, and 38 sequences from different regions of Brazil (initiated by BR): GO, Goiás state (Central), SP, São Paulo and RJ, Rio de Janeiro states (Southeast) and RS, Rio Grande do Sul state (South). Strains belonging to this study are marked with a black circle and reference sequences used were: 1a-m62321; 1b-m58335; 1c-d14853; 2a-d00944; 2b-d01221; 2c-d50409; 3a-d17763; 3b-d49374; 4a-y11604; 5a-y13184; 6a-y12083 and 6b-d84262. Genotype and subtype are indicated for each branch. The bootstrap values > 75% are indicated at the branches. The bar indicates genetic distance scale expressed as substitutions per 100 bases.

Among the anti-HCV-positive samples, eight were HCV RNA positive. The LiPA assay for the 5' NC region was able to determine the following genotypes: 1 (n = 3), 2 (n = 2), and 3 (n = 3). For subtyping analysis, all eight HCV RNA-positive samples could be amplified and sequenced in the NS5B region. Using phylogenetic tree analysis of the NS5B region (Figure 2), subtypes 1a (n = 1), 1b (n = 2), 2b (n = 2) and 3a (n = 3) were identified.

The present study represents the first report on the epidemiology of HCV infection in long-distance truck drivers in Brazil. The prevalence of HCV infection found was similar to that observed for the general population (1.42%-1.5%) [16,17]. Regarding data for truck drivers in other countries, the prevalence determined in this study was within the confidence interval range reported for Bangladesh (0.8%; 95% CI: 0.2-2.4), India (3.0%; 95% CI: 0.8-9.2), and Eritrea (6.0%; 95% CI: 1.5-17.5) [10-12], but it was lower than that observed in New Mexico, USA (8.5%; 95% CI: 6.5-11.0) [9].

Illicit drug use was the main predictor of HCV infection in the study population. Among the 81 truck drivers who reported illicit drug use, the majority had used non-injecting drugs, including the anti-HCV-positive individuals. The risk of HCV infection associated with non-injecting drugs is probably linked to the usual practice of sharing of non-injecting drug implements, such as pipes and straws, for smoking and sniffing/snorting drugs, which could lead to blood-to-blood contact [18]. Additionally, one-third of truck drivers in the current study reported regular use of amphetamines ("rebite", an oral stimulant), and most drivers also reported ongoing alcohol consumption. These habits are frequent among Brazilian truck drivers and appear to influence unsafe sex practices [6-8].

In fact, risk behaviors such as unprotected sex with multiple partners, including commercial sex workers, and history of STIs were frequent among the truck drivers investigated but were not associated with HCV infection. These data are consistent with findings obtained among truck drivers in New Mexico, USA [9].

Other variables related to percutaneous exposure to blood such as history of blood transfusion, sharing of personal hygiene tools, and HBV status were independent associated with HCV infection in the study population. Regarding blood transfusion, HCV infection was associated with this procedure before the introduction of screening for anti-HCV antibodies in Brazilian blood banks [4]. In fact, the anti-HCV-positive truck drivers reported having undergone blood transfusions with blood that had not been screened for anti-HCV antibodies (before November 1993). Similarly, but in lower level, the sharing of personal hygiene tools has also shown an association with HCV infection [19,20], as well as HBV status [9].

Among the nine anti-HCV-positive truck drivers, all but one were also HCV RNA positive, highlighting their potential to transmit HCV. Further, these individuals were infected with HCV genotypes 1, 2 and 3. These genotypes were also detected in other populations in Brazil, such as blood donors and chronically infected patients. The HCV subtypes identified in the study population (1a, 1b, 2b and 3a) are also consistent with the subtypes circulating in Brazil [15,21-23].

These results must be considered in the context of the study's limitations. The study population does not represent all long-distance truck drivers in Brazil but only those recruited at a large truck stop along the BR-153 highway in Central Brazil. Nonetheless, this is one of the longest and most important roads in Brazil, spanning the country from south to north, where long-distance truck drivers represent a highly mobile population. Moreover, given that there is a lack of available data on the serological and molecular epidemiology of HCV infection among long-distance truck drivers in Latin America, these data provide valuable insight into this topic.

In summary, the prevalence of HCV infection was similar to that observed for the general population. History of blood transfusion, sharing of personal hygiene tools, illicit drug use and HBV status were predictors of HCV infection. The HCV genotypes/subtypes identified in the study population are consistent with those circulating in Brazil.

## Competing interests

The authors declare that they have no competing interests.

## Authors' contributions

NRF, SAT, MAM, RMBM contributed to study concept and design. SAT was responsible for the statistical analysis. CLRL, NRSR, MPES, EL revised the manuscript for intellectual content. All authors read and approved the final version of the manuscript.
